# P-1730. Implementation of Pharmacist-Led Intervention on Oral Antibiotic Prescribing within Hospital Discharge Medication Reconciliation

**DOI:** 10.1093/ofid/ofae631.1894

**Published:** 2025-01-29

**Authors:** Amolee R Patel, Victoria Gavaghan, Thomas J Dilworth

**Affiliations:** Advocate Health Midwest, Milwaukee, Wisconsin; Advocate Health Care Midwest Region, Chicago, Illinois; AdvocateAuroraHealth, Aurora St. Luke’s Medical Center, milwaukee, Wisconsin

## Abstract

**Background:**

Inappropriate antibiotic exposure places patients at risk for collateral damage. Hospital discharge presents an opportunity to ensure antibiotic regimens represent an appropriate continuation of inpatient therapy and adhere to evidence-based activity spectrum and therapy duration recommendations. We sought to determine the impact of a pharmacist-led antibiotic stewardship intervention on oral antibiotic prescribing at hospital discharge, informed by institutional guidelines and targeted pharmacist education.

**Figure:**
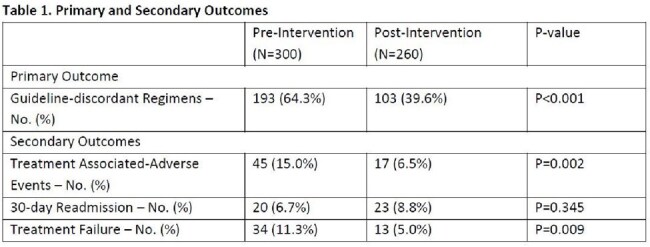

**Methods:**

This was a quasi-experimental study in which an antibiotic stewardship intervention was implemented within a 27-hospital, integrated health-system for patients discharging on oral antibiotics. Data following systemwide implementation (Jan-March 2023; POST) were compared to retrospective data without intervention (Aug-Sept 2022; PRE). The primary outcome measure was institutional guideline-discordant discharge regimens (agent, dose, duration of therapy). Secondary outcomes included treatment associated adverse-events, 30-day readmissions and treatment failure.

**Figure:**
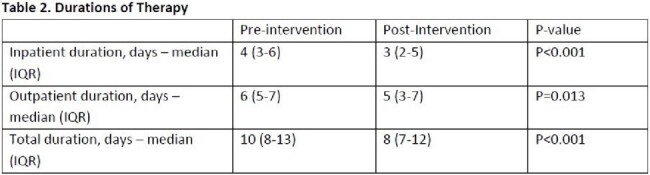

**Results:**

A total of 560 patients were included, 300 (53.6%) in PRE and 260 (46.4%) in POST. The most common antibiotic indications were community-acquired pneumonia (13.7%), urinary tract infection (26.8%) and skin and soft tissue infection (31.1%). The most frequent discordances included extended durations of therapy (47.7%), inappropriate targeted or empiric coverage (11.4%) and fluoroquinolone use in the setting of available alternatives (7.9%). Outcome results shown in Table 1. Pharmacists intervened upon 63 (24.2%) patients in POST with an 81.0% intervention acceptance rate. Rates of guideline-discordant regimens and treatment-associated adverse events were significantly lower in POST. Both median outpatient duration of therapy and total duration of therapy were significantly lower in POST as seen in Table 2.

**Conclusion:**

Hospital discharge is an important opportunity for antimicrobial stewardship programs. A pharmacist-led antibiotic stewardship initiative resulted in intervention on and optimization of guideline-discordant regimens. This initiative was also associated with both decreased antibiotic exposure and treatment-associated adverse events.

**Disclosures:**

**All Authors**: No reported disclosures

